# Epidermicin NI01 demonstrates potent *in vivo* activity in a murine model of methicillin-resistant *Staphylococcus aureus* skin infection

**DOI:** 10.1093/jacamr/dlag115

**Published:** 2026-06-18

**Authors:** Vicky Bennett, Gordon Barker, Andrew Sharp, Victoria L Birkett, Jennifer Williams, Pani Tourlomousis, Mathew Upton

**Affiliations:** Amprologix Ltd, Derriford Research Facility, Plymouth Science Park, 14 Research Way, Plymouth PL6 8BU, UK; Amprologix Ltd, Derriford Research Facility, Plymouth Science Park, 14 Research Way, Plymouth PL6 8BU, UK; Evotec (UK) Ltd, Alderley Park, Cheshire SK10 4TG, UK; Evotec (UK) Ltd, Alderley Park, Cheshire SK10 4TG, UK; Evotec (UK) Ltd, Alderley Park, Cheshire SK10 4TG, UK; Evotec (UK) Ltd, Alderley Park, Cheshire SK10 4TG, UK; Amprologix Ltd, Derriford Research Facility, Plymouth Science Park, 14 Research Way, Plymouth PL6 8BU, UK; School of Biomedical Sciences, University of Plymouth, Drake Circus, Plymouth PL4 8AA, UK

## Abstract

**Objectives:**

In the UK, over 16% of antibiotic prescriptions are for skin infections. New approaches are required to treat and prevent such infections, and new therapeutic modalities should be investigated. Epidermicin NI01 is a first-in-class bacteriocin with potent activity and a novel mode of action against MRSA and Streptococci, leading causes of community and healthcare acquired skin infections and a significant burden of antimicrobial resistance (AMR). The activity of NI01 in a model of MRSA skin infection was assessed during this study.

**Methods:**

Immunosuppressed male CD1 mice were wounded dorsally using tape-stripping and *S. aureus* strain USA300 was topically applied. Topical q24 h administration of vehicle (0.5% HMPC) or NI01 (4 dosing regimens in 0.5% HPMC) or 50 mg of Bactroban 2% (mupirocin) or Fucidin 2% (fusidic acid) was initiated at 24 h post-infection (hpi). At 96 hpi, infected skin was recovered for quantitative skin burden analysis.

**Results:**

There were no adverse effects observed due to infection or treatment. No reduction in skin burden was seen in the 3% NI01 mono-dose group. Treatments resulted in significant log_10_ reductions in cfu/g tissue of 2.82 (Fucidin), 2.48 (3% NI01 q24) and 2.47 (Bactroban). The MIC of NI01 for *S. aureus* strain USA300 was 4 mg/L before the study and *ex vivo*.

**Conclusions:**

These *in vivo* activity data warrant further development of NI01 for topical therapy of infections caused by drug-resistant priority pathogens. Clinical use could spare conventional antibiotics for serious and systemic infections and would support stewardship efforts to reduce mupirocin resistance in *S. aureus*.

## Introduction

Skin infections are among the most common reason for new primary care presentations in the UK,^1,2^ with antibiotic prescriptions for skin and wound infections representing >16% of all prescriptions.^3^ Many of these prescriptions are for topical mupirocin and fusidic acid to treat minor but prevalent conditions such as impetigo and infected eczema.^4^ The causative agents of these infections are predominantly Staphylococci or Streptococci^5,6^; however, both mupirocin and fusidic acid present with substantial resistance liabilities, as well as patient compliance issues due to prolonged treatment periods.^7,8^ Resistance rates for mupirocin and fusidic acid are growing globally, with up to 50% of clinical isolates in Europe, India, and USA showing resistance to either agent.^9–11^ This includes rising rates of mupirocin resistance in community settings due to the European epidemic fusidic acid-resistant impetigo clone in Belgium, with strains carrying genes encoding resistance to mupirocin (*mupA*) and fusidic acid (*fusB*), and the exfoliative toxin genes *eta* and *etb.*^7^ As mupirocin resistance is not routinely monitored, rates could be higher than reported in many geographies.^12^ Around 50% of impetigo patients experience recurrent infection within 12 months, necessitating repeated courses of antibiotics, which is significantly associated with the development of mupirocin resistance.^10,13^

Despite the wider implications, mupirocin also remains the leading standard of care for decolonization of MRSA carriage prior to elective procedures.^14^ Rising resistance therefore increases the risk of ineffective decolonization prior to surgery, and the prevalence of invasive MRSA infections. New therapeutic agents with rapid, potent activity that are effective with fewer doses would be preferable to current treatments for conditions like impetigo, could improve compliance in patients with skin infections,^8^ and could also contribute to improved outcomes for surgery patients.

Epidermicin NI01, an approximately 6000 Da bacteriocin derived from skin commensal *S. epidermidis*, has potent activity against Streptococci and Staphylococci, including isolates resistant to mupirocin, fusidic acid and other frontline topical or oral anti-infectives.^15^ We have demonstrated that epidermicin NI01 has excellent efficacy in an *in vivo* model of nasal decolonization of MRSA carriage.^16^ Based on this previous *in vitro* and *in vivo* data, we therefore suggest that NI01 has excellent potential for substantial impact as a therapy for many forms of skin infection and prevention of devastating invasive sequelae that may occur. Impetigo is well suited to topical therapy as this enables targeted delivery to the infection site, and improves adherence to therapy through avoidance of systemic delivery and an easier administration route for caregivers, which are particular concerns when treating paediatric patients.^8,12^ Introduction of epidermicin NI01 and other novel agents for topical skin therapy could help preserve the use of small molecule drugs for treatment of these more serious infections.

This study sought to evaluate the *in vivo* efficacy of epidermicin NI01 in a model of wounded skin infection. We selected *S*. *aureus* strain NRS 384 for this study given the role of the USA 300 clone in community acquired skin infection.^17^ Firstly, different inoculum levels of *S*. *aureus* NRS 384 were used to identify the most suitable level for efficacy studies. Then, the efficacy of epidermicin NI01 (hereafter NI01) was assessed when applied topically at different doses and frequencies against *S. aureus* NRS 384 in a four-day mouse model of superficial skin infection. The activity of NI01 was compared with that of fusidic acid and mupirocin, which are antimicrobials indicated for the treatment of impetigo in the UK, according to NICE guidelines.^18^

## Materials and methods

### Ethics

All animal experiments were performed at the Evotec (UK) facility, Alderley Park, under UK Home Office Licence PP7817737, with local ethical committee clearance. The Evotec (UK) animal facility holds a UK Home Office Establishment Licence, is fully AAALAC-I accredited and is a dedicated Containment Level 2 facility.

### Bacterial strains and growth conditions

MRSA strain NRS 384 (USA 300 clone^19^) and *S. aureus* ATCC 29213 (included as a quality control strain for antimicrobial susceptibility testing) were recovered from −80°C storage in glycerol bead stocks onto Mannitol Salt Agar (MSA; Oxoid). To obtain single colonies for the quantification of colony forming units (cfu) in samples, preparations were serially diluted in PBS and plated on MSA, followed by incubation at 37°C for 18–24 h, after which colonies were counted. For all infection experiments, *S. aureus* NRS 384 was cultured in Mueller Hinton Broth (MHB; Becton-Dickinson) to mid-logarithmic phase in a shaking incubator at 300 rpm and 37°C. Cell pellets were harvested via centrifugation, washed once with PBS, then diluted to the appropriate inoculum concentration using PBS. The inoculum was prepared based on a calibration curve generated to correlate optical density with viable counts (cfu/mL), with the exact viability of each inoculum determined by serial dilution and plating on MSA, followed by incubation at 37°C for 18–24 h before colonies were counted.

### Preparation of topical treatments

NI01 was manufactured and supplied in lyophilized form as an acetate salt by Almac Ltd, and stored at −20°C until use. Hydroxypropylmethylcellulose (HPMC; Merck) was prepared at a concentration of 0.5% in sterile water, filter sterilized, and stored at 4°C prior to use as the vehicle for NI01 solutions. On the day of administration, NI01 was formulated to final solutions of 0.5%, 1%, or 3% in 0.5% HPMC and stored at 4°C prior to use and between doses. The reference compounds mupirocin (Bactroban 2% cream; GSK) and fusidic acid (Fucidin 20 mg/g cream; Leo Laboratories) were administered as comparators for *in vivo* efficacy and were also stored at 4°C between doses.

### Antimicrobial susceptibility testing

Antimicrobial susceptibility testing was performed using the broth microdilution method according to Clinical and Laboratory Standards Institute (CLSI) guidelines M7-A12 and M100-S34, with *S. aureus* ATCC 29213 and levofloxacin included as quality control standards in each assay. NI01 was prepared in PBS, mupirocin (Merck KGaA, Darmstadt, Germany, Lot. 0000144223) was prepared in DMSO, and fusidic acid (Merck KGaA, Darmstadt, Germany, Lot. MKBX9355 V) and levofloxacin (Merck KGaA, Darmstadt, Germany, Lot. 038M4848 V) were both prepared in water. All drugs were supplied as lyophilized powder. Bacterial inocula were diluted in cation adjusted MHB to provide a final bacterial density of 2–8 × 10^5 ^cfu/mL, then dispensed into 96-well plates containing 2-fold serial dilutions of each antimicrobial agent. Plates were incubated aerobically at 37°C for 18 h, after which time the MIC was determined as the lowest concentration of each test article that completely inhibited visible bacterial growth.

### Animal strain and housing

Male CD-1 specific pathogen-free mice were supplied by Charles River Laboratories (Margate, UK). Mice were 11–15 g on arrival and were allowed to acclimatize for at least seven days prior to use. Body weight was recorded daily, and weight loss relative to day 0 calculated over the course of the study to ensure mice did not exceed clinical limits. The clinical condition of mice was observed and scored regularly throughout the study to monitor animal welfare. Any mice reaching clinical endpoints were to be euthanized with an overdose of pentobarbitone administered intraperitoneally without further delay.

### Preparation of wound site and infection with MRSA

Neutropenia was induced by subcutaneous administration of 150 mg/kg cyclophosphamide (Baxter) 4 days prior to infection, and 100 mg/kg 1 day prior to infection. Mice were anesthetised with ketamine/medetomidine (Orion Pharma UK) and maintained on heat controlled mats throughout the infection procedure to prevent hypothermia. The dorsal skin area was prepared by shaving, followed by depilation (Veet) to ensure complete hair removal, after which residual depilation cream was removed by rinsing with warm water. Once the skin was fully dried, a central 2 cm2 section was marked and tape-stripped three times with fresh zinc oxide tape (Leukoplast) for each application.

A 10 µL aliquot of bacterial inoculum equivalent to 2 × 10^6 ^cfu/mouse was applied to the centre of the marked area and allowed to absorb for 5 min. Any remaining bacterial suspension was evenly spread across the marked section using a sterile loop and allowed to absorb completely. Anaesthesia was reversed by administration of atipamezole (Orion Pharma UK), after which mice were returned to their home cages and placed in a warming cabinet to recover for at least 1 h under continuous observation, with wet food provided to assist recovery.

### Determination of bacterial skin burden after treatment with NI01

At 24 h post-infection, mice were randomly assigned to one of seven different treatment groups (*n* = 6 mice per group) and were subject to isoflurane anaesthesia. Three treatment groups received NI01 administered once daily for three consecutive days, as three doses of 3%, three doses of 1%, or three doses of 0.5% (W/V). A fourth group received NI01 as a single 3% dose. Two comparator groups were treated with either mupirocin or fusidic acid (both at 2%) and a final vehicle control group was treated with 0.5% HPMC. Both comparator groups and the vehicle control group were treated once daily for three consecutive days. Compounds were spread evenly over the infected area at a volume of 50 µL, or at a mass of 50 mg for the comparator creams.

Mice were weighed and clinically assessed daily. The clinical condition of all animals was assessed prior to humane euthanasia with an overdose of pentobarbitone administered intraperitoneally at 96 h post-infection. The central 2 cm2 skin infection area was excised from the underlying subcutis and cross sectioned into two halves. The superior half from each mouse was collected, weighed, then homogenized in 1 mL cold sterile PBS containing 10% glycerol via bead beating with a Precellys homogeniser (Bertin Technologies, Montigny-le-Bretonneux, France). Tissue homogenates were serially diluted and plated on MSA followed by incubation at 37°C for 18–24 h before colonies were counted.

### Data and statistical analysis

All bacterial burden data were log_10_ transformed prior to analysis. Terminal bacterial burden in each of the treatment groups was compared to the vehicle control using one-way ANOVA with Dunnett’s post-hoc test. To assess changes in terminal bacterial burden from the baseline, log_10_ fold-change in cfu/g was calculated for each group, where baseline was defined as the cfu/g at 4-h post-inoculation, calculated as part of pilot studies to determine appropriate inoculum size (Table S1, available as Supplementary data at *JAC-AMR* Online). The log_10_-fold change for each group was compared to zero (no change from baseline) using one-sample *t*-tests with Bonferroni correction for multiple comparisons. All statistical analyses were performed using GraphPad Prism version 10.4.0.

## Results

### Comparison of the *in vitro* efficacy of NI01 and the reference antibiotics

To establish the *in vitro* antimicrobial activity of NI01 compared to that of the reference compounds mupirocin and fusidic acid, the MIC for each compound against *S*. *aureus* NRS 384 was determined. NI01 exhibited an MIC of 4 mg/L, while fusidic acid and mupirocin demonstrated higher *in vitro* potency, at 0.5 mg/L (Table 1).

**Table 1.** MICs of NI01 and comparator antibiotics for *S. aureus* NRS 384

Isolate	Source	MIC (mg/L)			
		NI01	Levofloxacin	Mupirocin	Fusidic acid
Pre-infection parental isolate	Freezer stock	4	8	0.5	0.5
Post-infection isolate	Vehicle-treated mouse	4	4–8	—	—
Post-infection isolate	NI01-treated mouse	4	4	—	—

Pre-infection data represent modal values from ≥3 biological replicates. Post-infection isolates were obtained from vehicle treated (*n* = 2) and NI01 treated (*n* = 2) mice, with one isolate tested per animal. -, not tested.

### NI01 demonstrates efficacy in a mouse model of superficial skin infection with *S*. *aureus* strain NRS 384

**Figure 1.**
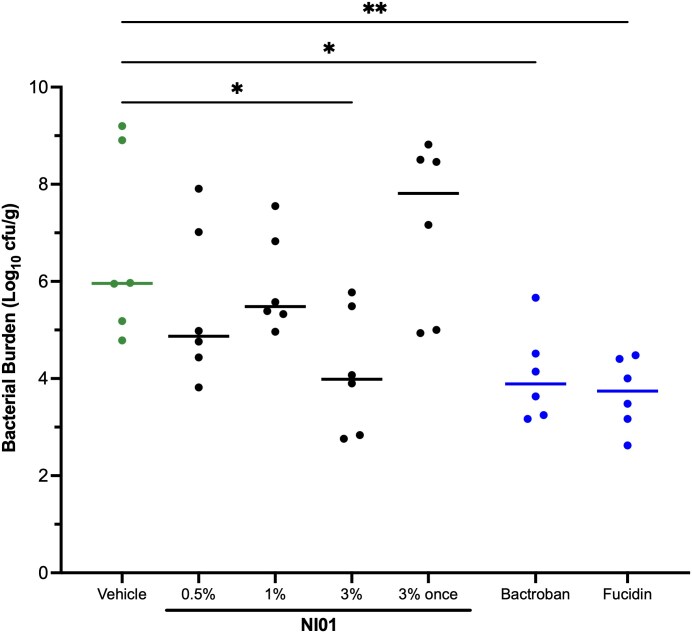
Impact of NI01 treatment on bacterial burden of mouse skin following *S. aureus* infection. Male CD-1 specific pathogen-free mice (*n* = 6 per treatment group) were infected with *S. aureus* NRS 384 at 2.6 × 10^6 ^cfu/mouse. After 24 h, mice were treated once daily for three days with either 0.5% HPMC vehicle, NI01 (0.5%, 1% or 3%), 2% Bactroban, or 2% Fucidin. An additional group received a single dose of 3% NI01 on the first day only. The bacterial burden after 96 h was compared to the vehicle control group using one-way ANOVA with Dunnett’s post-hoc test. *P < 0.05; **P < 0.01. cfu, colony forming units; HPMC, hydroxypropylmethylcellulose.

At 96 h post-infection, terminal bacterial burdens for NI01 0.5% q24 h and NI01 1% q24 h were 5.49 ± 1.60 log_10 _cfu/g (1.18 log_10_ reduction compared to vehicle) and 5.94 ± 1.02 log_10 _cfu/g (0.73 log_10_ reduction compared with vehicle) respectively, neither of which were statistically significant. A single dose of NI01 3% did not reduce terminal bacterial burden, with a terminal burden of 7.15 ± 1.78 log10 cfu/g (0.48 log_10_ increase compared to vehicle; *P* > 0.05). Of the NI01 treatments, only NI01 3% administered q24 h resulted in a significant reduction in bacterial burden compared to the vehicle control group (terminal bacterial burden 4.14 ± 1.28 log_10 _cfu/g, representing a 2.53 log_10_ reduction; *P* = 0.016). These findings represent a dose dependent effect of NI01 *in vivo*, with maximal efficacy achieved with a 3% q24 h dose (Figure 1).

The efficacy of 3% NI01 q24 h was equivalent to that of the standard-of-care product Bactroban (terminal burden 4.06 ± 0.94 cfu/g, 2.61 log_10_ reduction; *P* = 0.013) The other standard-of-care treatment Fucidin exhibited the greatest reduction (terminal burden 3.69 ± 0.733 cfu/g, 2.98 log_10_ reduction; *P* = 0.004). Intragroup variability was noted across all treatment groups, and may reflect the fact that neutrophil numbers begin to increase three days after cyclophosphamide-induced neutropenia.^20^ This could impact microbial clearance, and lead to the variability seen at the 96 h end point. All raw data are shown in Table S2.

### Impact of HPMC vehicle on bacterial burden

**Figure 2.**
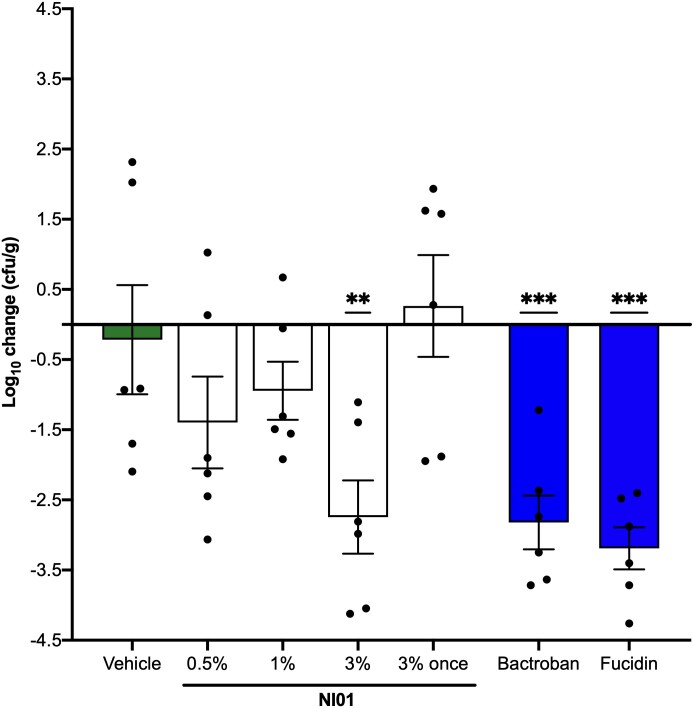
Change in bacterial burden compared to baseline across treatment groups. The log_10_ change in tissue burden of *S. aureus* NRS 384 at 96 h relative to 4 h baseline was determined for each treatment group and the vehicle control. Data represent the mean ± SD (*n* = 6 per group) with individual replicates shown. A log_10_ change of zero is equivalent to no change in bacterial burden. Comparisons to baseline were made using one-sample *t*-tests with Bonferroni correction. **P < 0.007; ***P < 0.001.

To confirm that the HPMC vehicle lacked intrinsic antimicrobial activity and that efficacy seen was occurring due to NI01 activity alone, changes in terminal bacterial burden for all groups were calculated relative to the baseline inoculum, determined as bacterial burden at 4 h post-infection (Figure 2). There was no significant change from baseline for the vehicle control group (0.217 log_10_ reduction; *P* = 0.792). This indicates that while the HPMC vehicle prevented significant proliferation, it lacked meaningful antimicrobial activity *in vivo*. Antimicrobial efficacy observed in NI01 treatment groups is therefore due to NI01 exposure alone. Both Bactroban and Fucidin exhibited significant reductions from the baseline, although the relative contributions of active ingredients and excipients used in their formulation to the overall efficacy cannot be determined from the results obtained in this study.

### Impact of prolonged NI01 exposure on antimicrobial susceptibility of *S. aureus* NRS 384

To determine whether prolonged *in vivo* exposure to NI01 could induce any changes in susceptibility, *S. aureus* isolates were recovered from mouse tissue at 96 h post-infection and MICs were determined. Isolates were obtained from mice treated with either vehicle (mice 1 and 5, *n* = 2) or 3% NI01 q24 h (mice 19 and 23, *n* = 2). For all recovered isolates, the MIC of NI01 remained consistent with the parental strain (4 mg/L; Table 1). The MICs for the levofloxacin control also showed no significant change compared to the parental isolate. One isolate from a vehicle exposed mouse had a levofloxacin MIC at 96 h of 8 mg/L compared to the parental strain MIC of 4 mg/L; however, this is within a 2-fold dilution range and was therefore deemed a non-significant change (Table 1). These findings indicate that prolonged *in vivo* exposure to NI01 did not alter susceptibility under the conditions tested.

## Discussion

There is an acknowledged need for new agents for use in the prevention and treatment of skin infections. Agents for topical use with novel modalities could bring benefits for patients and improve stewardship of conventional antibiotics by helping to preserve their use as important systemic antimicrobials and reduce selection pressure for AMR. While previous work has demonstrated the ability of NI01 to protect against MRSA infection in *Galleria mellonella,*^21^ as well as efficacy in nasal decolonization,^16^ this current study highlights the potential for NI01 to be used a therapeutic by demonstrating efficacy in a skin infection model. Initiation of treatment at 24 h post-infection indicates NI01 can be effective as a therapeutic, rather than a purely prophylactic agent. This indicates its clinical relevance for use in common skin conditions, and supports the continued clinical development of NI01 as a topical antimicrobial.

In this study, topical administration of 3% NI01 q24 h over 3 days demonstrated significant antimicrobial efficacy against *S. aureus* NRS 384 relative to vehicle-treated controls. Notably, NI01 achieved efficacy comparable to Bactroban, the current standard-of-care antibiotic ointment containing 2% mupirocin, despite substantial differences in formulation complexity and active ingredient concentration. While 3% NI01 contains ∼500 μmol NI01 per 100 g, equivalent masses of both Bactroban 2% and Fucidin 20 mg/g contain approximately 4000 μmol of either mupirocin or fusidic acid, respectively. The 3% NI01 formulation therefore demonstrates equivalent efficacy to Bactroban at ∼8-fold lower molar concentration, indicating a potentially higher intrinsic potency. In addition, NI01 was formulated solely in an HPMC vehicle, which was found to possess no intrinsic antimicrobial activity. This confirms that the observed bacterial burden reduction resulted exclusively from NI01 activity. In contrast, Bactroban is a fully formulated commercial product containing multiple excipients, several of which possess documented antimicrobial properties that may contribute to overall product efficacy.^12,22^ While this study design did not permit quantification of the relative contributions of mupirocin versus formulation excipients to Bactroban's efficacy, the achievement of comparable performance by NI01 in a basic HPMC vehicle is encouraging at this early development stage, and there is significant opportunity for formulation optimization and enhanced efficacy.

Considering the rising rates of resistance to both fusidic acid and mupirocin, it is also encouraging that in this study, *in vivo* exposure to NI01 for 3 days did not alter *in vitro* susceptibility for recovered isolates compared to the parental strain. In contrast, resistance to fusidic acid has previously been reported after topical therapy during a similar murine model of MRSA infection, where isolates from mice treated with Fucidin demonstrated increased fusidic acid MIC values compared to the parental isolate when subsequently tested *in vitro.*^23^ Although the short duration and small sample size in this study mean definitive predictions on the propensity of resistance development to NI01 cannot be made, ongoing serial passage experiments and comparisons across multiple clinical isolates will provide a more comprehensive assessment of this risk. Considering the data presented here together with evidence from previous *in vitro* work^15^ and its complex mode of action,^24^ we are optimistic that topical use of NI01 is less likely to lead to the rapid resistance issues that often compromise the action of conventional small molecule antibiotics.

### Conclusions

NI01 has demonstrated efficacy against MRSA in two rodent models of acute localized infection: the cotton rat nasal infection model^16^ and the neutropenic mouse model of superficial skin infection, described here. NI01 was well tolerated, with no toxicity concerns at the concentrations used, and demonstrated equivalent efficacy to the current standard-of care treatment Bactroban. A follow-up dose-response study targeting higher dose levels could more precisely define the therapeutic range of NI01, as could studies with an optimized formulation for delivery to the skin.

## Supplementary Material

dlag115_Supplementary_Data

## Supplementary data

Tables S1 and S2 are available as Supplementary data at JAC-AMR Online.
