# Supplementary material for: Epidermicin NI01 demonstrates potent in vivo activity in a murine model of methicillin-resistant Staphylococcus aureus skin infection
Source: JAC Antimicrob Resist. 2026 Jun 18;8(3):dlag115. doi: 10.1093/jacamr/dlag115 (PMC13278623; doi:10.1093/jacamr/dlag115)
Supplement: dlag115_Supplementary_Data [file dlag115_supplementary_data.docx]

**Table S1: Individual skin burden data for animals included in pilot study to identify optimal inoculum level for *in vivo* efficacy studies**

| **Group** | **2x10^8^ 4hpi** | **2x10^8^ 72hpi** | **2x10^7^ 4hpi** | **2x10^7^ 72hpi** | **2x10^6^ 4hpi** | **2x10^6^ 72hpi** |
| --- | --- | --- | --- | --- | --- | --- |
| **Skin burden**  **(Log_10_ CFU/g)** | 9.50 | 8.35 | 7.46 | 9.47 | 6.88 | 8.93 |
|  | 8.65 | 9.61 | 7.88 | 8.92 | 6.88 | 9.99 |

A pilot study tested escalating *S. aureus* inocula to determine an appropriate inoculum density for the main study. Four mice were inoculated at each density (cfu/mouse), with two euthanised at 4hpi, and two euthanised at 72hpi for quantification of bacterial burden. Mean bacterial burden was then calculated from the two mice at each time point. Mean bacterial burden at 4hpi for the 2x10^6^ cfu/mouse inoculum was used as baseline bacterial burden for further analysis in the main study. hpi: hours post infection.

**Table S2: Individual skin burden data for animals included in the *in vivo* efficacy study of MRSA wounded skin infection**

| **Group** | **Vehicle q24** | **NI01 5 mg/ml (0.5%) q24** | **NI01 10 mg/ml (1%) q24** | **NI01 30 mg/ml (3%) q24** | **NI01 30 mg/ml (3%) ONCE** | **Bactroban 2% q24** | **Fucidin 2% q24** |
| --- | --- | --- | --- | --- | --- | --- | --- |
| **Skin burden**  **(Log_10_ CFU/g)** | 5.95 | 7.02 | 7.55 | 5.49 | 8.82 | 5.66 | 3.48 |
|  | 5.97 | 4.76 | 5.32 | 2.76 | 8.51 | 4.52 | 3.17 |
|  | 8.91 | 4.44 | 4.96 | 4.07 | 7.16 | 3.63 | 2.62 |
|  | 4.79 | 3.82 | 6.83 | 2.84 | 8.46 | 3.25 | 4.40 |
|  | 5.19 | 4.98 | 5.39 | 5.77 | 4.94 | 3.17 | 4.00 |
|  | 9.2 | 7.91 | 5.58 | 3.9 | 5.00 | 4.14 | 4.48 |
